# Health status by gender, hair color, and eye color: Red-haired women are the most divergent

**DOI:** 10.1371/journal.pone.0190238

**Published:** 2017-12-28

**Authors:** Peter Frost, Karel Kleisner, Jaroslav Flegr

**Affiliations:** 1 Department of Anthropology, Université Laval, Quebec City, Canada; 2 Division of Biology, Faculty of Science, Charles University, Prague, Viničná 7, Czech Republic; 3 Division of Applied Neurosciences and Brain Imagination, National Institute of Mental Health, Klecany, Czech Republic; Macquarie University, AUSTRALIA

## Abstract

Red hair is associated in women with pain sensitivity. This medical condition, and perhaps others, seems facilitated by the combination of being red-haired and female. We tested this hypothesis by questioning a large sample of Czech and Slovak respondents about the natural redness and darkness of their hair, their natural eye color, their physical and mental health (24 categories), and other personal attributes (height, weight, number of children, lifelong number of sexual partners, frequency of smoking). Red-haired women did worse than other women in ten health categories and better in only three, being particularly prone to colorectal, cervical, uterine, and ovarian cancer. Red-haired men showed a balanced pattern, doing better than other men in three health categories and worse in three. Number of children was the only category where both male and female redheads did better than other respondents. We also confirmed earlier findings that red hair is naturally more frequent in women than in men. Of the ‘new’ hair and eye colors, red hair diverges the most from the ancestral state of black hair and brown eyes, being the most sexually dimorphic variant not only in population frequency but also in health status. This divergent health status may have one or more causes: direct effects of red hair pigments (pheomelanins) or their by-products; effects of other genes that show linkage with genes involved in pheomelanin production; excessive prenatal exposure to estrogen (which facilitates expression of red hair during fetal development and which, at high levels, may cause health problems later in life); evolutionary recentness of red hair and corresponding lack of time to correct negative side effects; or genetic incompatibilities associated with the allele Val92Met, which seems to be of Neanderthal origin and is one of the alleles that can cause red hair.

## Introduction

It has long been known that redheads are at higher risk of sunburn and skin cancer. This is to be expected because red hair is associated with fair skin, which is more vulnerable to UV radiation [1]. Less expectedly, red hair is also associated with pain sensitivity, endometriosis, Parkinson’s disease, decreased platelet function and, perhaps, defects in the immune system [2–11]. These associations seem to involve a risk factor not directly related to fairness of skin and vulnerability to UV.

The risk factor seems to be specific to women. Pain sensitivity is higher in female redheads than in male redheads [2]. Endometriosis is associated with the combination of being a female redhead and having no history of infertility [6]. In the case of Parkinson’s disease, red hair alleles seem to compromise the integrity of dopaminergic neurons, but no one has determined whether red-haired women are affected more than red-haired men [9]. In the case of decreased platelet function, the association with red hair has been investigated only in women [10]. If a female-specific factor is interacting with red hair to facilitate these medical conditions, a plausible candidate may be higher levels of estrogen in the fetal environment. Prenatal estrogen influences not only the emergence of certain medical conditions later in life but also the development of certain hair and eye colors, particularly red hair. According to a twin study, women are more likely than men to have red hair even when the genotype is the same [12]. Prenatal estrogen may also affect eye color, since face shape is more feminized in blue-eyed men than in brown-eyed men of the same ethnic background [13,14]. In addition to favoring blue eyes over brown eyes, prenatal estrogen seems to favor green eyes over blue eyes, the so-called blue-eye genotype being expressed in women more often as green eyes [15]. In sum, there seems to be a general tendency for women to exhibit less frequent hair and eye colors at the expense of more frequent ones [16,17].

Prenatal estrogen may therefore mediate the relationship between red hair and certain aspects of health, including some that remain unsuspected. It was only by chance that researchers discovered the three-way association between being a woman, having red hair, and feeling more sensitivity to pain. There has been no systematic effort to identify all female-specific associations between human health and red hair, let alone between human health and each of the different hair and eye colors.

For these reasons, we wished to find out how different aspects of human health vary as a function of hair/eye color. We also wished to see how well the variance is explained by the two known risk factors: 1) vulnerability to UV, as measured by relative importance of skin cancer; and 2) gender, specifically being a woman. To this end, we used an existing dataset collected for an unrelated purpose: a survey on the RhD factor in relation to various health categories in a Czech and Slovak population. This survey encompassed a very large number of individuals and could thus capture relatively weak associations between health status and other factors.

## Methods

### Respondents and recruitment

The present study reanalyzed data originally collected for a survey on the RhD factor in relation to human health. Respondents were recruited by a Facebook-based snowball method [18], as described by a previous paper [19]. In short, potential volunteers were invited to participate in “research to investigate how blood groups and other biological factors relate to personality, performance, morphology, and health.” The invitation was posted on the Facebook wall page “Guinea Pigs” (in Czech “Pokusni kralici”) for Czech and Slovak nationals willing to take part in evolutionary psychology experiments (www.facebook.com/pokusnikralici) [20]. The first page of the electronic questionnaire described the goals of the study. The following note was included: “The questionnaire is anonymous and obtained data will be used exclusively for scientific purposes. Your cooperation with the project is voluntary, and you can terminate it at any time by exiting this website.” The first and final pages of the questionnaire both had a Facebook share button and the following request: “We need the highest possible number of respondents. Therefore, please share the link to this questionnaire with your friends, for example on Facebook.” The share button was pressed by 575 respondents, with the result that we finally obtained data from 7,044 Czechs and Slovaks between 28/4/2014 and 12/09/2016.

All participants provided informed consent by pressing the corresponding button on the electronic form. All methods were performed in accordance with the relevant guidelines and regulations. The study, including the method of obtaining informed consent (by pressing the Next button on the first page), was approved by the IRB of the Faculty of Science, No. 2014/21."

### Questionnaire

The questionnaire was distributed as a Czech/English Qualtrics survey (http://1url.cz/q05K). In the first part of the questionnaire, respondents were asked, among other questions, to rate the natural darkness of their hair and eyes and the natural redness of their hair on two 6-point Likert scales (on the first scale, 0 = light hair/eyes and 5 = very dark hair/eyes; on the second scale, 0 = hair not at all red and 5 = completely red). They were also asked to choose the natural color of their eyes from a list of eight colors (blue, green, brown, black, grey, amber, hazel, yellow). Hair/eye color was thus self-reported and should be understood as such throughout this paper. Finally, they were asked about their body height and weight, number of children, lifelong number of sexual partners, and how often they smoked (0- never, 1- maximum of once per month, 2- maximum of once per week, 3- maximum of once per day, 4- several times a day, 5- more than 20 cigarettes a day, 6- more than 40 cigarettes a day).

The medical part of the questionnaire was prepared by two physicians: a clinician (internist/hematologist), and a researcher (molecular geneticist). Questions fell into two parts, one using subjective measures of health status and the other more objective measures. Respondents were first asked to rate the presence and intensity of their health problems on a 6-point Likert scale. These questions were on physical health and mental health in general and on more specific health categories: allergies; cancer; digestion; fertility; genitourinary system; heart and vascular system; hematology; immune system; metabolism, including endocrine system; musculoskeletal system; nervous system; respiratory organs; sense organs; and sexual function. The second part of the questionnaire was designed to provide objective information on health status. For example, respondents were asked how many physician-prescribed drugs they were currently taking per day, how many “different herbs, food supplements, multivitamins, superfoods etc.” they were currently taking per day, and how often they had used antibiotics during the past 365 days.

As a benchmark for the relative strength of associations between hair/eye color and the 24 health categories, we measured the associations between these categories and two unrelated but well-known risk factors: body mass index (BMI) and smoking.

For some of these categories, we also asked the respondents to state the specific disorders they had or used to have. For the ‘Cancer’ category, respondents were asked “What kind of cancer are you suffering from or have you suffered from?” They then read a list of disorders and ticked the appropriate boxes.

### Data analysis

Statistica v. 10 and IBM SPSS v. 21 were used for most of the statistical analysis. MANCOVAs (with gender, eye color, or hair color as predictors) were performed by the “adonis” function available within Vegan package in R [21]. Differences in age were assessed by t-tests. Chi^2^ tests were used to compare the frequencies of eye colors in men and women. Relationships between hair/eye color and gender or age were analyzed by both nonparametric (partial Kendall Tau correlation with age or gender as a covariate) and parametric tests using general linear models with gender, age, and gender*age interaction as independent variables. Both categories of tests produced very similar results, and therefore only the results of the more conservative nonparametric tests are reported. Logistic regression (Quasi-Newton estimation method) was used for the analysis of relation between cancer and hair redness and the hair redness*gender interaction. Other ordinal and binary data were analyzed by a partial Kendall Tau correlation test, which is used to measure the strength and significance of associations between binary, ordinal, or continuous data regardless of their distributions and which can be used to control for one confounding variable, here the respondent’s age [22,23]. To compute partial Kendall Tau and the significance of each variable, after controlling for age, we used an Excel spreadsheet available at: http://web.natur.cuni.cz/flegr/programy.php. Because many disorders tend to be more common in men than in women or vice versa, associations between the health categories and the hair/eye colors were always analyzed separately for men and women. Whenever fewer than 10 respondents reported a disorder, a Fisher exact test was used to determine the significance of the association between hair/eye color and each health category. To correct for multiple tests, and the associated increase in the false discovery rate, we used the Benjamini-Hochberg procedure with the false discovery rate pre-set to 0.25 [24]. In contrast to the Bonferroni correction, this procedure takes into account the distribution of p values of performed multiple tests. Therefore, when the factor under study has multiple effects, the number of significant results after the correction may be higher than before the correction (Here, and throughout this paper, the term “effect” does not imply a direct relationship of cause and effect). To measure eye color diversity, we used the Simpson index λ, which was computed as λ = SUMA(p_i_)^2^, where p_1_–p_i_ denotes proportions of respondents with 1-i eye color in the population under study [25]. This index reflects the probability of two randomly-selected respondents carrying the same character, here the same eye color.

## Results

### Characteristics of respondents

Information on hair/eye color was provided by 2,558 men and 4,472 women out of 7,044 Czech and Slovak respondents (the others did not complete the questionnaire part of the test). Mean age was higher for the men (36.8, std. dev. 13.5) than for the women (34.6, std. dev. 13.0) t_7028_ = 6.9, p < 0.0005.

**Fig 1:**
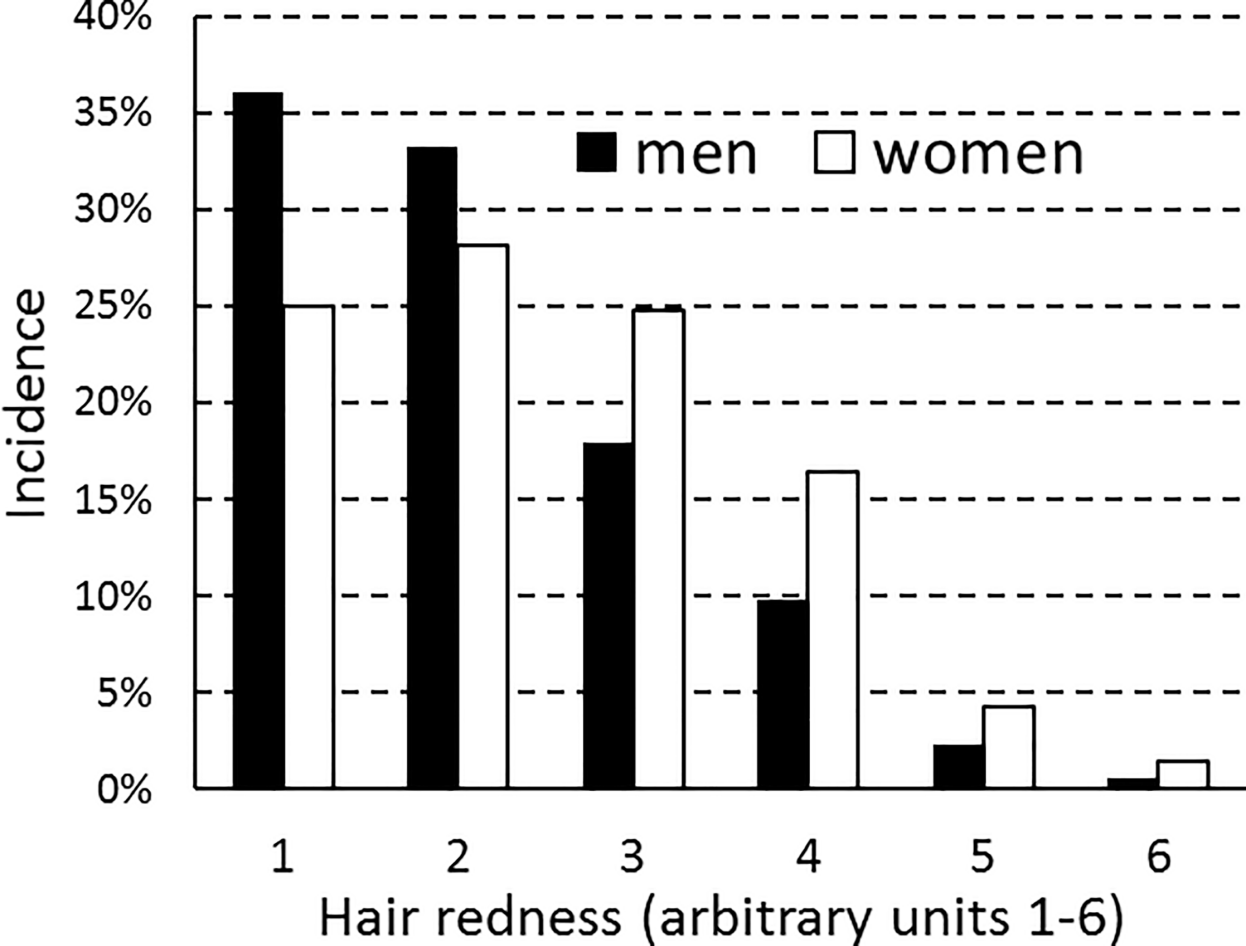
Gradations of hair redness: Population frequencies for men and women. Respondents rated hair redness on a scale of 1 to 6 where 1 = not at all red and 6 = completely red.

**Fig 2:**
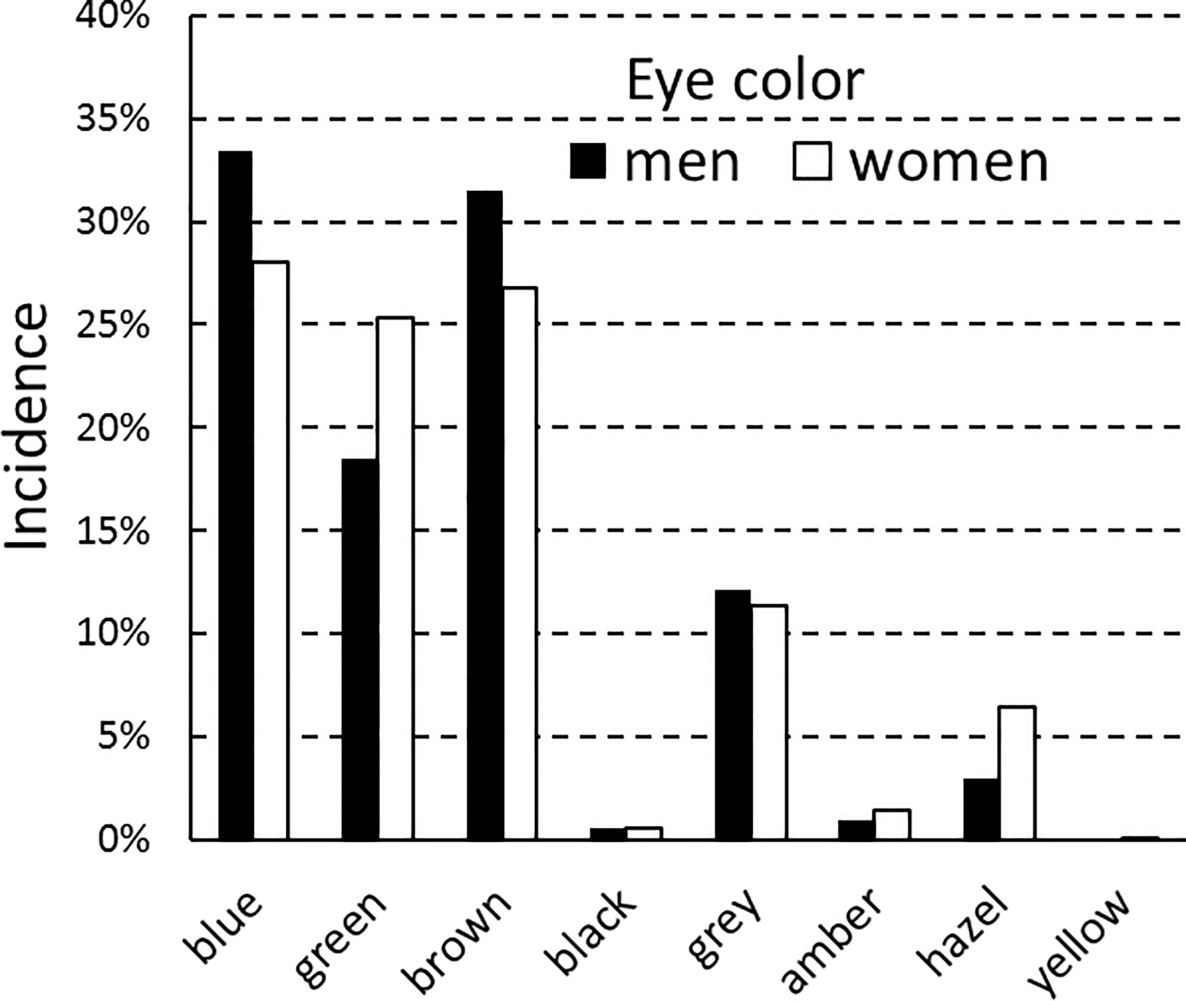
Eye colors: Population frequencies for men and women.

Figs 1 and 2 show how different gradations of hair redness and eye darkness were distributed among male and female respondents. In keeping with the findings of an earlier twin study, red hair was more frequent in women than in men [12]. Hair tended to be lighter in women than in men, but eyes were equally dark. A closer look at the data showed that eye color was more diverse in women than in men, with green eyes being more frequent in women and blue and brown eyes more frequent in men. This gender difference is seen in a higher Simpson index for men (0.263) than for women (0.234). Women had higher eye-color diversity in all 5-year age groups, except for the 41–45 age group.

Age was associated in women with darker eyes, darker hair, and redder hair (Table 1), and in men with redder hair. After controlling for age, we still found gender differences: male hair was significantly darker (mean 4.07 vs. 3.87, p < 0.0005) and less red (mean 2.09 vs. 2.51, p < 0.0005). No gender difference was observed in eye darkness (mean 3.39 vs, 3.42, p = 0.56). These results are consistent with earlier findings: hair darkens with age until midlife but male hair darkens more rapidly, with the result that male hair is darker than female hair from about 17 years old onward [26]. The respondents were too young to exhibit the lightening of hair color that generally comes with late adulthood.

**Table 1:** Correlations between eye/hair color and age.

	Hair darkness	Hair redness	Eye darkness	Age (men)	Age (women)
Hair darkness		**-0.18**	**0.38**	0.00	**0.07**
Hair redness	-0.01		**-0.08**	**0.14**	**0.18**
Eye darkness	**0.39**	0.00		0.02	**0.03**

The upper right of the table (excluding the last two columns) shows partial Kendall Tau correlations (age controlled) for men, and the lower left the same results for women. The last two columns show standard Kendall Tau correlations between hair/eye color and age in men and women, respectively. Significant correlations are in bold.

### Associations between health categories and hair/eye color

We looked for significant associations between 24 health categories and different hair/eye colors. Yellow eye color was reported by only 5 respondents and therefore excluded from the analyses. The results, shown in Table 2 for men and in Table 3 for women, suggest that women have more of the negative medical conditions associated with hair or eye color. Red hair in particular seems associated in women with the largest number of negative conditions and the fewest positive ones. To measure the strength of these associations we performed MANCOVAs (multivariate analyses of variance) on those factors found to be significant, using respondent age as the covariate. For each health category, the power of red hair to explain variance in health status was compared with that of BMI and that of smoking. By order of importance, red-haired women were prone to disorders in the following health categories: (1) Musculoskeletal; (2) Heart & vascular, Cancer, Fertility; (3) Metabolic; (4) Sexual function; (5) Genitourinary. For women in these categories, red hair seemed comparable to BMI and smoking in its power to explain variance in health status.

**Table 2:** Results for men: Associations with eye color, hair color, BMI, and smoking.

	Blue	Green	Brown	Black	Grey	Amber	Hazel	Eye darkness	Hair darkness	Hair redness	BMI	Smoking
Physical health problems in general	-0.015	-0.019	0.011	**0.033**	**0.041**	**-0.030**	**-0.025**	0.004	0.015	**-0.031**	**0.172**	**0.093**
Mental health problems in general	**-0.026**	0.009	0.014	0.014	0.015	0.003	**-0.024**	0.011	-0.024	**0.032**	**-0.023**	**0.083**
Specific health problems:												
Antibiotics/ year	0.007	-0.001	-0.004	**-0.032**	0.002	**-0.036**	**0.026**	-0.018	-0.002	0.006	0.014	**-0.050**
Acute care/ 5 years	0.008	**-0.034**	**0.048**	-0.006	**-0.034**	-0.004	-0.007	0.026	0.014	-0.008	0.011	**-0.034**
Different med. specialist seen every 2 years	0.002	-0.003	0.002	0.026	0.002	-0.015	-0.012	-0.001	-0.021	0.017	**0.083**	**-0.030**
Number of prescription drugs/day	0.015	0.023	-0.014	**0.051**	-0.017	-0.012	**-0.039**	-0.016	-0.026	**0.031**	**0.133**	**-0.053**
Number of alternative medicines/day	0.021	0.000	-0.019	-0.020	0.001	-0.015	0.007	-0.003	-0.010	**0.032**	0.013	**-0.049**
Allergies	-0.001	0.003	-0.004	-0.014	0.015	0.020	**-0.028**	-0.012	0.015	**-0.053**	-0.001	**-0.037**
Immunological	**0.024**	**-0.051**	0.019	-0.003	0.023	-0.023	**-0.037**	-0.005	-0.003	0.000	-0.015	**-0.034**
Digestive	**-0.033**	0.018	0.004	0.025	**0.034**	0.001	**-0.040**	0.020	-0.017	0.012	**0.023**	**0.053**
Heart & vascular system	-0.022	0.012	-0.007	0.003	**0.037**	**0.040**	**-0.044**	0.003	0.005	-0.008	**0.076**	**0.025**
Hematological	0.018	0.018	-0.015	-0.023	0.003	-0.011	**-0.042**	-0.028	-0.017	0.024	-0.002	-0.009
Metabolic	0.018	-0.013	-0.001	0.013	0.012	-0.009	**-0.042**	-0.007	-0.024	0.022	**0.163**	-0.017
Cancer	0.013	-0.030	-0.015	-0.010	**0.026**	**0.053**	-0.004	0.000	-0.020	0.018	**0.039**	**-0.019**
Fertility	-0.015	-0.023	0.012	**0.074**	**0.028**	0.012	**-0.036**	0.006	0.000	-0.003	0.017	0.001
Genitourinary	0.016	-0.008	-0.004	-0.024	-0.009	0.001	0.013	-0.013	-0.025	0.018	-0.011	-0.028
Sense organs	**-0.034**	-0.002	0.025	-0.005	**0.027**	-0.004	-0.018	0.023	0.005	0.004	0.017	0.009
Neurological	**-0.028**	-0.023	0.007	0.003	**0.059**	-0.012	-0.001	0.008	0.005	0.023	-0.018	-0.012
Psychiatric	**-0.037**	0.001	0.014	**0.042**	0.007	0.002	**0.030**	0.027	0.008	0.008	-0.009	**0.095**
Sexual function	-0.003	0.003	-0.011	0.020	**0.029**	-0.006	**-0.032**	-0.022	-0.011	0.007	**0.034**	**0.074**
Musculoskeletal	**-0.052**	-0.025	**0.063**	0.014	0.016	0.010	-0.017	**0.066**	0.001	0.026	**0.037**	**-0.021**
Respiratory	-0.011	-0.013	0.030	0.019	-0.006	0.010	**-0.027**	0.014	0.006	0.001	**0.045**	**0.063**
Tiredness (frequency)	**-0.034**	0.024	0.027	**0.038**	-0.009	-0.008	**-0.027**	0.025	0.009	-0.011	0.008	**0.096**
Headaches (frequency)	**-0.036**	-0.009	0.023	0.020	0.017	**0.032**	-0.002	0.010	0.010	-0.011	0.008	**0.068**
Reproductive/ mating success:												
Number of children	**0.023**	-0.007	-0.026	**-0.035**	0.018	0.014	-0.006	-0.025	-0.007	**0.039**	**0.099**	**-0.030**
Number of sexual partners	0.018	-0.002	0.020	0.025	**-0.046**	0.000	**-0.019**	-0.005	-0.003	0.009	**0.069**	**0.182**
Negative divergences in health status	1	0	2	6	9	3	3	1	0	3	10	10
Positive divergences in health status	9	2	0	1	1	2	13	0	0	3	3	10

The figures (age-controlled partial Kendall Tau correlations) show the strength and direction of associations between variables on the top and on the left. A positive figure means a positive association between a respondent characteristic (column headings) and a category of human health, including number of children and sexual partners (row headings). Associations that remain significant after correction for multiple testing are in bold. The last two rows show the total number of significant associations where the divergence in health status is either negative or positive. A higher number of children and a higher number of sexual partners are classified as positive divergences in health status.

**Table 3:** Results for women: Associations with eye color, hair color, BMI, and smoking.

	Blue	Green	Brown	Black	Grey	Amber	Hazel	Eye darkness	Hair darkness	Hair redness	BMI	Smoking
Physical health problems in general	-0.007	**-0.024**	**0.039**	**-0.027**	0.010	-0.020	-0.009	0.011	**0.020**	**0.020**	**0.228**	**0.054**
Mental health problems in general	**-0.033**	0.014	0.013	**-0.024**	**0.019**	0.001	-0.007	0.003	**0.020**	**0.047**	0.013	**0.041**
Specific health problems:												
Antibiotics/ year	-0.005	**-0.026**	**0.020**	0.012	0.015	-0.005	-0.001	0.002	0.013	**-0.035**	**0.045**	**0.027**
Acute care/ 5 years	**-0.025**	-0.013	**0.035**	0.015	0.010	-0.007	-0.009	**0.019**	**0.025**	0.002	**0.047**	**0.029**
Different med. specialist seen every 2 years	-0.018	-0.014	**0.046**	0.006	-0.005	-0.022	-0.014	**0.023**	0.014	-0.006	**0.052**	0.013
Number of prescription drugs/day	**-0.055**	**-0.023**	**0.077**	-0.009	**0.021**	0.002	**-0.028**	**0.045**	**0.023**	0.008	**0.122**	0.005
Number of alternative medicines/day	0.001	0.012	0.003	0.008	-0.012	-0.022	0.001	0.007	0.004	0.013	**-0.019**	-0.019
Allergies	**-0.039**	-0.001	**0.049**	0.011	-0.011	-0.001	-0.002	**0.034**	0.016	-0.012	**0.030**	**-0.025**
Immunological	**-0.020**	-0.015	**0.034**	**0.040**	**-0.020**	0.019	0.006	**0.039**	**0.039**	0.007	**0.020**	**0.018**
Digestive	-0.015	0.018	0.016	0.012	-0.002	0.010	**-0.037**	0.014	-0.001	0.008	**0.031**	0.009
Heart & vascular system	-0.010	0.009	0.005	-0.010	0.011	-0.006	**-0.019**	0.002	0.015	**0.044**	**0.040**	**0.017**
Hematological	0.004	-0.016	0.000	**0.036**	0.008	-0.014	0.009	-0.004	0.000	0.007	-0.006	-0.007
Metabolic	**-0.041**	**-0.022**	**0.039**	0.019	0.010	0.021	0.014	**0.038**	**0.023**	**0.045**	**0.187**	0.007
Cancer	0.012	**0.024**	-0.014	-0.012	**-0.027**	0.017	-0.010	0.003	-0.009	**0.067**	0.011	0.013
Fertility	**-0.030**	0.005	0.015	-0.005	0.013	0.007	0.001	0.013	0.015	**0.042**	0.006	-0.010
Genitourinary	-0.017	-0.007	**0.033**	-0.006	**0.019**	-0.024	**-0.025**	0.016	0.009	**0.028**	**-0.026**	0.013
Sense organs	-0.002	-0.007	-0.016	**-0.034**	**0.038**	0.005	0.003	**-0.029**	0.000	0.011	**0.041**	**-0.024**
Neurological	-0.013	-0.003	0.006	**0.023**	**0.021**	-0.001	**-0.020**	-0.002	0.011	0.014	0.011	-0.005
Psychiatric	**-0.033**	**0.025**	**-0.020**	-0.012	**0.035**	0.004	0.005	-0.006	0.006	**0.020**	0.007	**0.098**
Sexual function	-0.012	0.003	0.012	-0.016	0.016	0.006	**-0.024**	-0.002	0.015	**0.030**	0.017	**0.016**
Musculoskeletal	0.000	0.010	-0.005	-0.007	0.005	-0.015	-0.005	-0.004	-0.018	**0.053**	**0.040**	0.013
Respiratory	**-0.026**	0.006	**0.027**	**0.026**	-0.006	-0.008	-0.008	**0.026**	0.013	-0.006	**0.084**	**0.053**
Tiredness (frequency)	-0.004	0.005	0.001	0.003	0.016	-0.007	**-0.022**	-0.005	0.014	0.014	**0.021**	**0.058**
Headaches (frequency)	-0.001	0.006	-0.004	0.013	0.010	-0.009	**-0.019**	-0.009	0.015	-0.002	0.014	**0.019**
Reproductive/ mating success:												
Number of children	0.006	0.004	**0.026**	-0.008	-0.016	-0.010	**-0.041**	**0.018**	**0.030**	**0.143**	**0.096**	**-0.027**
Number of sexual partners	0.010	0.016	-0.006	0.014	**-0.027**	0.014	-0.008	-0.001	**-0.021**	**0.061**	0.011	**0.246**
Negative divergences in health status	0	2	10	4	7	0	1	7	7	10	14	12
Positive divergences in health status	9	4	2	3	2	0	8	2	1	3	3	3

See Table 2 legend.

To determine the relative importance of skin cancer in the Cancer category, we looked at the incidences of specific types of cancer. The results are shown in Table 4. Red-haired men were thus more prone to colorectal cancer, while red-haired women were more prone to colorectal cancer, precancerous cervical or uterine lesions, cervical or uterine cancer, ovarian cancer, and other types of cancer. This higher cancer risk was not due to a higher rate of skin cancer, which was only non-significantly more frequent in red-haired men (OR = 1.52) and women (OR = 1.43).

**Table 4:** Differences in cancer rate between redheads and non-redheads by sex and by specific type of cancer.

Type of cancer	Men						Women					
	Red- Can-	Red-Can+	Red+ Can-	Red+ Can+	OR	p	Red-Can-	Red-Can+	Red+ Can-	Red+ Can+	OR	p
Esophageal cancer	1762	0	268	0			2946	1	836	1	3.52	0.394
Stomach cancer	1762	0	268	0			2946	1	836	1	3.52	0.394
Colorectal cancer	1762	0	264	4	273.56	**0.000**	2947	0	834	3	109.53	**0.011**
Liver cancer	1762	0	267	1	72.57	0.132	2947	0	836	1	38.77	0.221
Lung cancer	1761	1	267	1	6.59	0.247	2947	0	837	0		
Melanoma, other skin cancers	1753	9	266	2	1.52	0.271	2937	10	833	4	1.43	0.243
Breast cancer	1762	0	268	0			2932	15	830	7	1.66	0.318
Cervical uterine precancerosis	1677	0	259	0			2723	64	756	27	1.52	**0.008**
Cervical uterine cancer	1762	0	268	0			2902	45	817	20	1.58	**0.003**
Corpus uteri cancer	1762	0	268	0			2940	7	835	2	1.04	1.000
Ovarian cancer	1762	0	268	0			2942	5	832	5	3.54	**0.015**
Prostate cancer	1753	9	266	2	1.52	0.184	2947	0	837	0		
Lymphoma, myeloma multiple	1760	2	268	0	0.31	1.000	2947	0	837	0		
Leukemia	1758	4	268	0	0.16	1.000	2941	6	836	1	0.63	1.000
Bladder cancer	1761	1	268	0	0.60	1.000	2945	2	836	1	1.85	0.528
Mouth, oropharynx cancers	1761	1	268	0	0.60	1.000	2947	0	836	1	38.77	0.221
Adeno-carcinoma	1762	0	268	0			2943	4	837	0	0.09	0.582
Papilloma cancer	1762	0	268	0			2941	6	833	4	2.37	0.422
Other types of cancer	1747	15	265	3	1.35	0.642	2929	18	825	12	2.37	**0.000**

Red- Can- = Number of non-redheads (i.e., respondents whose intensity of redness is 1–3 on a 6-point Likert scale) without the specific type of cancer

Red- Can+ = Number of non-redheads with the specific type of cancer

Red+ Can- = Number of redheads (i.e., respondents whose intensity of redness is 4–6) without the specific type of cancer

Red+ Can+ = Number of redheads with the specific type of cancer

Odds Ratios (OR) and statistical significance (p) respectively are shown for men and women and for each specific type of cancer. Age was controlled by performing a partial Kendall Tau’s correlation whenever the incidence of a specific type of cancer exceeded 9. Otherwise, a Fisher’s exact test was performed to determine statistical significance. ORs higher than 1 indicate that redness is positively associated with the incidence of the specific type of cancer. Results are in bold if significant in two-sided tests after Benjamini-Hochberg correction for multiple tests, and p-values < 0.0005 are coded as 0.000.

### Interactions with gender

Because divergences in health status differed between men and women, particularly among red-haired respondents, we looked for a significant interaction with gender. To this end, we first performed four MANCOVAs to see whether variance in respondent health correlated significantly with gender, eye darkness, hair darkness, and hair redness. We then performed three MANCOVAs to see whether variance in respondent health correlated significantly with an interaction between gender and any of the other variables: eye darkness, hair darkness, or hair redness. Finally, we constructed a new binary variable—presence or absence of green eyes—and performed two MANCOVAs to see whether variance in respondent health correlated significantly with this new variable or with an interaction between it and gender. All MANCOVAs had respondent age as the covariate. The results are shown in Table 5. Although respondent health correlated significantly with gender, hair redness, and eye darkness taken separately, and although female respondents differed significantly from male respondents in hair redness and hair darkness (but not in eye darkness), there were no significant three-way interactions between gender, respondent health, and any of the above variables for hair or eye color (Table 5). In the case of eye color, a linear regression on eye darkness may not be the best approach if the relationship between gender and eye color cannot be described simply in terms of eye darkness. As we have seen, women are less likely than men to be blue-eyed or brown-eyed, while conversely being more often green-eyed (Fig 2). This was why we constructed the binary variable of green eyes versus all other eye colors, and we found that an interaction between this factor and gender significantly explained some of the variance in respondent health. In general, green-eyed women were healthier than the other respondents, except for a greater propensity to have cancer and psychiatric problems.

**Table 5:** Correlations of all divergences in health status with gender, hair color, or eye color.

Independent variable	R	p-value
gender	0.12	0.001***
hair redness	0.04	0.001***
hair darkness	0.02	0.35
eye darkness	0.03	0.002**
gender*hair redness	0.02	0.182
gender*hair darkness	0.01	0.583
gender*eye darkness	0.02	0.405
green eyes vs. all other eye colors	0.02	0.049*
gender*green eyes vs. all other eye colors	0.12	0.001***

**Fig 3:**
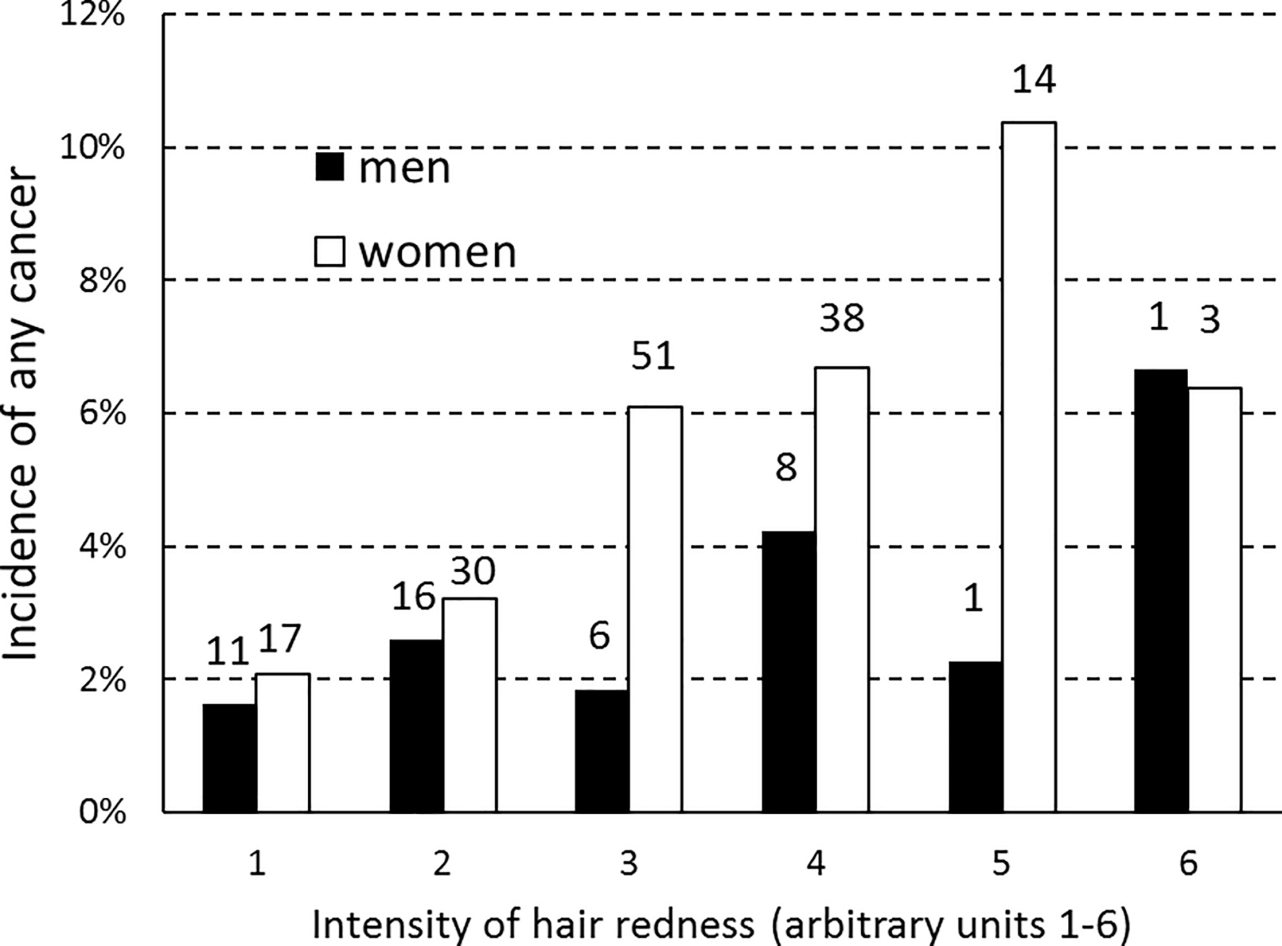
Incidence of any cancer by gradation of hair redness, for men and women. The numbers above the columns show the numbers of respondents in each category.

This finding made us take a second look at the relationship between female respondent health and hair redness. That relationship, too, might not be fully understood through a linear regression. We specifically looked at the cancer data because the relationship between negative health status and hair redness was strongest in that category. We performed a logistic regression with incidence of any cancer as the dependent variable (0 = no cancer reported, 1 = cancer or precancerous lesion reported) and with three independent variables: gender, age, hair redness, and gender*hair redness interaction. For women only, incidence of any cancer was significantly associated with hair redness (OR range = 3.99, p<0.0001) and age (OR range = 12.1, p<0.0001). For men only, it was significantly associated with age (OR range = 50.7, p<0.0001) but not with hair redness (OR range = 1.63, p = 0.457). For men and women together, it was significantly associated with age (OR range = 17.7, p<0.0001) and hair redness (OR range = 3.74, p<0.0001) but not with gender (OR range = 0.67, p = 0.331) or gender*hair redness interaction (OR range = 0.47, p = 0.381). The results are shown in Fig 3.

**Fig 4:**
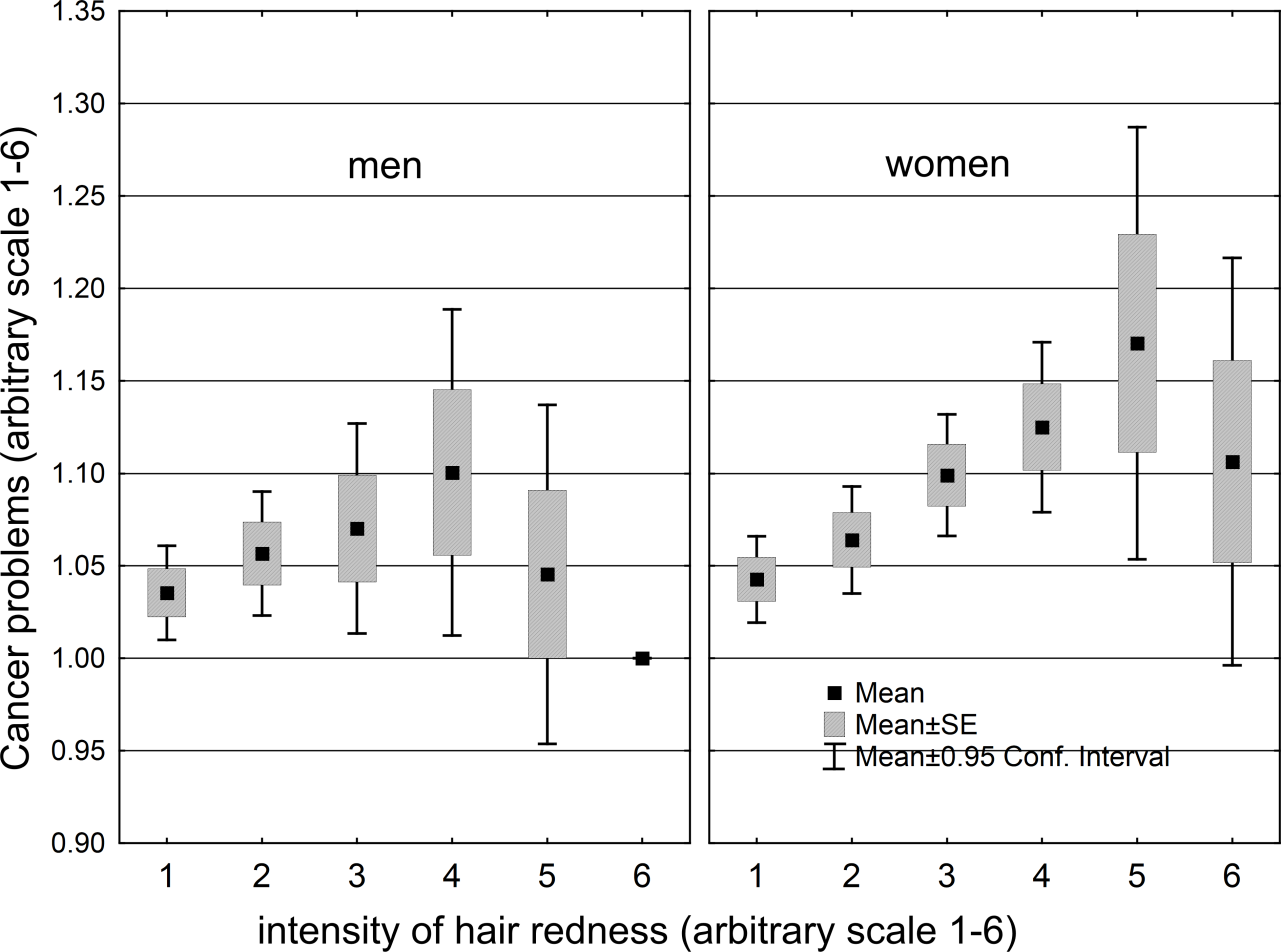
Mean seriousness of cancer by gradation of hair redness, for men and women.

The gender difference was greatest at the next-to-last gradation of hair redness. To learn more about this interaction between gender and gradation of hair redness, we plotted the reported mean seriousness of cancer (where 1 = no cancer reported and 6 = very serious problem with cancer) as a function of hair redness. This new variable may provide a clearer picture because it contains more information than simply the presence or absence of cancer. The results are shown in Fig 4. Mean seriousness of cancer increased steadily with increasing hair redness up to a certain gradation of redness and then decreased. This relationship was stronger in women than in men and peaked at a higher gradation of redness in women than in men. For women only, mean seriousness of cancer was significantly associated with age (p<0.0005) and hair redness (p = 0.014). For men only, it was significantly associated with age (p<0.0005) but not with hair redness (p = 0.719). For men and women together, it was significantly associated with age (p<0.005) and hair redness (p = 0.0005) but not with gender (p = 0.438) or gender*hair redness interaction (p = 0.292).

## Discussion

Red hair seems to be costly for women’s health. In this study, red-haired women did worse than other women in ten health categories and better in only three. In general, women incurred more costs and gained fewer benefits from red hair than from any other hair or eye color. Brown eyes held second place but were associated with smaller negative or positive divergences in health status than those associated with red hair. Red-haired men showed a balanced pattern, doing better than other men in three health categories and worse in three. Number of children was the only category where both male and female redheads did better than non-redheads. In terms of reproductive and, ultimately, evolutionary success, red hair seems to be a plus rather than a minus.

The cancer rate was higher among red-haired women than among other women, and we initially suspected a higher rate of skin cancer as the cause. A closer look at the data, however, showed that the higher cancer rate was due not to a higher incidence of skin cancer but rather to a higher incidence of cancers in the colorectal region, the cervix, the uterus, and the ovaries (Table 4). Because estrogen influences the development of the last three organs from the fetal stage onward, the higher cancer rate may be better explained by a higher level of prenatal exposure to estrogen, rather than by increased vulnerability to UV. This explanation is supported by the higher incidence among red-haired women of osteoporosis and obstetric complications (results not shown), both of which are either more frequent in women or specific to women. It may seem surprising that the skin cancer rate was only slightly higher for redheads than for non-redheads, given the many studies that point to red hair as a risk factor. Such studies, however, generally concern countries like the United States and Australia, whose citizens are exposed to a higher intensity of UV because they live at lower latitudes than do Czech citizens and also because a higher proportion of them have regularly traveled to tropical or subtropical resorts for the past half-century or longer.

As for the other divergences in health status associated with red hair, they too are not easily attributable to fairness of skin, and hence to UV vulnerability, again because they were reported mainly by female respondents. Although women are fairer-skinned than men, this sexual dimorphism is relatively small in fair-skinned humans and in redheads in particular, among whom both sexes are pushed up against the physiological ‘ceiling’ of skin reflectance [27, 28]. Moreover, if the cause were fairer skin and a consequently increased vulnerability to UV, we would expect to see a similar pattern with blue eyes, which are likewise associated with fair skin [1]. Yet, relative to other hair and eye colors in our sample, blue eyes were associated with fewer costs to male or female health, while providing women with the highest total of benefits and men the second-highest.

Some of our findings are consistent with previous findings in the literature. Despite having more children on average, the red-haired women of this study had a higher incidence of fertility problems, which would be consistent with the higher incidence of endometriosis reported in previous studies. They also had more neurological problems, although none of these involved Parkinson’s disease. Actually, few cases of Parkinson’s would be expected, given the relatively young age of the respondents. Red-haired women showed no obvious indications of increased pain sensitivity in this study, although in some cases they might have reported more medical problems because sensitivity to pain had made them seek medical assistance more readily.

These divergences in health status thus seem to be due to a female-specific factor that is most strongly expressed in red-haired women. The relationship between this factor and hair redness seems curvilinear, i.e., female health progressively worsens on average with redder gradations of hair, but only up to a certain point. If we take the data on seriousness of cancer, the worst health status was reported by women with the next-to-last gradation of hair redness. Those with the reddest hair were actually somewhat better off (Fig 3); however, this category had only three women and one man. With respect to eye color, the female-specific factor seemed to act more strongly on women with green eyes than on those with brown or blue eyes. Red hair and green eyes thus exhibit a pattern of gender difference that seems to mirror the effects of estrogen on development of hair and eye color. Red hair is more frequent in women than in men (Fig 1); similarly, green eyes are more frequent in women than in men, with brown eyes and blue eyes showing the reverse pattern (Fig 2).

It seems, then, that some hair and eye colors are associated with a more divergent health status, particularly in women. Red-haired women exhibit the most divergences, including a previously unreported vulnerability to colorectal, ovarian, and cervical cancer. Not all of the divergences are for the worse. In particular, red-haired women seem to enjoy greater reproductive and mating success, as measured by number of children and number of sexual partners. It may be that they have more children because they begin having them at an earlier age, although a recent study has reported that red-haired men and women lose their virginity at a later age on average [29]. An alternative explanation is that red-haired women attract not only more sexual partners but also better sexual partners who can support a larger family. This greater attractiveness of red hair might be due to its being less common than other hair colors. It has been argued that European hair and eye colors, including red hair, have coexisted in a dynamic equilibrium where each color variant is sexually attractive in proportion to its scarcity. If a color variant becomes too common, it loses this novelty value, and the pressure of selection shifts to less common ones [16, 17]. In the case of red hair, the increased susceptibility to certain medical conditions tends to depress its population frequency below the frequency it would have if sexual attractiveness were the only selection pressure. The gap between this second equilibrium and the first may explain the relative popularity of red-haired women: they are never sufficiently common to lose their novelty value.

What causes this divergent health status in red-haired women? The causation can be framed in either biochemical or evolutionary terms. First, in terms of biochemical causation, there may be a direct effect by red hair pigments (pheomelanins) or their by-products. There may also be direct effects by other genes that show linkage with genes involved in pheomelanin production. Nonetheless, such a causation would not explain why certain medical conditions occur more often in red-haired women than in red-haired men. As argued in the Introduction, the female-specific factor may be prenatal estrogen, i.e., the same factor that promotes the expression of red hair in the female fetus and brings about the higher frequency of this hair color in women. In the womb, estrogen levels are nearer the top end of the normal range for fetal development if the fetus is a female who ends up with red hair. The risk of later health problems is therefore proportionately greater.

Second, in terms of evolutionary causation, red hair may have been the last hair color to emerge in modern humans; therefore, not enough time has passed for corrective evolution, either through new alleles that produce red hair with fewer side effects or through modifier genes that neutralize the side effects of existing red hair alleles. This situation is typical of rapid evolution over relatively short spans of time [30]. Another possible scenario is that red hair alleles emerged among Neanderthals and then introgressed into early modern Europeans when the two groups coexisted in Europe. Such introgression could cause genetic incompatibilities and thus explain the increased susceptibility to certain medical conditions. Red hair is produced mainly by five loss-of-function alleles at the *MCIR* gene, and a recent study has identified one of them, Val92Met, as a likely Neanderthal introgression. The same study, however, found that the four other alleles are not of Neanderthal origin [31]. Given that modern humans entered Europe c. 45,000 BP and reached northern Europe c. 30,000 BP, and that the Neanderthals went extinct c. 40,000 BP, the scenario of Neanderthal introgression makes sense for Val92Met, which is found throughout Eurasia. However, the other loss-of-function alleles, which are more specific to northern Europe, probably originated in modern humans.

Before proceeding to the conclusion, we should acknowledge three limitations of the present study. First, the hair and eye color data were subjective, being self-report. Less healthy individuals might have a tendency to exaggerate the redness of their hair. Second, a rather small number of respondents had the highest intensity of hair redness. To chart health problems as a function of hair redness, particularly for its highest intensity, it will be necessary to repeat our study with a larger population or one with a higher frequency of red hair. Third, the data had a high level of noise because of the subjective nature of the questions and because criteria for self-rating varied from one individual to another. As a result, even when significant correlations were found between respondent health and different factors (gender, age, hair redness, hair darkness, eye darkness, etc.), they could not explain more than a tiny proportion of total variance in health status among the respondents. We should emphasize that this tininess at least partly reflects noise in the data and does not indicate the relative importance of these factors. To provide a point of comparison, we examined respondent data on BMI and smoking, both of which strongly affect human health. Using the data in Table 3 (significant negative divergences in women’s health status), we found that, on average, BMI explained twice as much variance in these medical conditions as did red hair (τ = 0.071, vs. 0.040), while smoking explained approximately the same amount (τ = 0.039). Thus, BMI and smoking were comparable to red hair in their power to explain variance in women’s health for these conditions.

To conclude, our findings may shed light not only on the health risks associated with red hair but also on the evolution of this highly visible color trait and, more generally, on how the diverse European palette of hair and eye colors came into being. This evolution seems to have occurred for the most part in relatively recent times, probably no earlier than the entry of modern humans into northern Europe some 30,000 years ago and no later than the oldest DNA evidence of red hair, blond hair, and blue/green eyes (Motala, Sweden), which has been dated to some 8,000 years ago [32]. The short time span (< 1000 generations) suggests that some form of selection, possibly sexual selection, was driving this diversification of hair and eye colors in early Europeans. Of these ‘new’ color variants, red hair seems to diverge the most from the ancestral state of black hair and brown eyes. It is the most sexually dimorphic variant, not only in population frequency but also in associated medical conditions.
